# Adiponectin levels in people with Latent Autoimmune Diabetes-a case control study

**DOI:** 10.1186/1756-0500-3-317

**Published:** 2010-11-22

**Authors:** Sinead Brophy, Helen Davies, Jeffrey W Stephens, Sarah L Prior, Mark Atkinson, Stephen Bain, Rhys Williams

**Affiliations:** 1 Senior Lecturer in Epidemiology and Public Health. College of Medicine, Swansea University. Swansea, SA2 8PP UK; 2 College of Medicine, Swansea University, Swansea, SA2 8PP UK

## Abstract

**Background:**

To examine adiponectin levels in people with Latent Autoimmune Diabetes in Adults using a matched pair case control study.

**Findings:**

Patients with LADA (n = 64), were matched for sex with type 2 diabetic and non-diabetic controls. A matched paired T-test was used to examine average adiponectin levels in the LADA patients' versus controls. The average adiponectin level for the LADA patients was 9.96 μg/ml compared to 6.4 μg/ml for Type 2 matched controls and 9.6 μg/ml for non-diabetic controls. Mean difference for the LADA-type 2 comparison was calculated after data was log transformed and showed a difference of 1.58 μg/ml (95%CI: 1.28-1.95, p = 0.0001). There was no significant difference between LADA and non-diabetic controls (p = 0.54).

**Conclusions:**

Adiponectin levels are higher among people with LADA compared to those with type 2 diabetes and are equivalent to levels seen in non-diabetic controls. This suggests that risk of complications in LADA, as with type 1 diabetes may be related more to glycaemic control rather than to factors of the metabolic syndrome.

## Background

Adiponectin is a marker of insulin resistance, with lower levels being associated with reduced insulin sensitivity, less favourable lipid profiles and increased risk of developing cardiovascular disease in many studies [1-4]. However this may not be so clear cut as more recent studies in coronary patients have yielded contradictory results [5]. Furthermore, adiponectin levels are lower in people with type 2 diabetes and the metabolic syndrome [6]. However, adiponectin levels have been found to be elevated in people with a type 1 diabetes [7] and may be associated with increased onset of microalbuminuria [8]. Latent autoimmune diabetes in adults [LADA] is a slowly progressive type 1 diabetes [9-12] with features of both type 1 and type 2 diabetes. A person with LADA is not insulin dependent at diagnosis and in this way resembles type 2 diabetes. LADA can be distinguished from type 2 diabetes by antibody test such as a glutamic acid decarboxylase antibody (GADA) test. There is very limited information available regarding the risk of heart disease among people with LADA and existing data is contradictory [13-15]. Adiponectin levels are a reflection of the amount of visceral fat or the obesity levels of the person. Thus, adiponectin levels may give an indication of the extent to which abdominal obesity may play a role in risk of complications for LADA patients. This study aims to examine Adiponectin levels in people with LADA compared to those with type 2 diabetes and compared to non-diabetic controls.

## Methods

### Design

Matched case control study with cases (LADA patients) matched with controls (type 2 diabetes and non-diabetic controls) for sex as adiponectin levels are higher in women compared to men [16]. The cases were matched one to one with a type 2 diabetes control and a non-diabetic control. Cases were not matched for BMI, as obesity was not believed to be a confounder but to be a causal factor [17].

### Patients

1,630 patients over the age of 18, with a diagnosis of type 2 diabetes within the previous 12 months (not requiring insulin at diagnosis) were tested for GADA within the Immunology Laboratory of the ABM University NHS Trust. Of these patients, 64 (3.9%) who had GADA titres of 20 WHO units (27 Female, 37 Male) or more, were recorded as GADA positive and labelled as LADA cases. Those patients had a GADA test of 5 WHO units or less were recorded as GADA negative and labelled as type 2 control patients. Those with GADA titres between 5 and 20 WHO units were excluded from the study.

Sex matched controls (n = 64) were selected at random from the GADA negative type 2 patients. In addition, sex matched anonymised blood samples were obtained from people who did not have a diagnosis of diabetes on their medical record (non-diabetic controls (n = 64)) and had a blood test which had a negative result were selected from normal blood samples going through the Biochemistry Department of the ABM University NHS Trust.

### Setting

GADA testing was conducted in May 2006 to Dec 2008. Patients were tested by their general practitioner (GP) at the same time as undergoing their first diabetes annual review (DAR) blood tests.

### Adiponection

The concentration of plasma adiponectin was assayed using an ELISA-based Quantikine Human Acrp30 immunoassay kit (R&D Systems, Minneapolis, Minnesota) according to the manufacturer's instructions. The assay measures total (low, middle and high molecular weight) adiponectin and employs a quantitative sandwich enzyme immunoassay technique to determine adiponectin concentrations. Standards over the range of 1-250 mg/L were prepared using recombinant human adiponectin. All plasma samples were diluted according to the manufacturer's instructions. The intra-and inter-assay coefficients of variation are 5.83% and 3.12% respectively.

### GADA antibody testing

GADA were measured using an ELISA (65 kDa antibody) from RSR Cardiff, UK [18] and analysed in the Department of Immunology at Singleton Hospital, Abertawe Bromorganwwg University NHS Trust. This ELISA kit achieved 98% (n = 100) specificity and 92% (n = 50) sensitivity in the Diabetes Antibody Standardization Programme (DASP) [19].

### Statistical analysis

The Shapiro-Wilk test was used to test for normal distribution. The data were log transformed and matched pair T-tests were used to compare LADA patients with type 2 diabetic controls and with non-diabetic controls. SPSS version 13 was used for analysis.

Ethical approval was granted by the South West Wales REC in October 2008.

## Results

The clinical characteristics and demographics of LADA patients and the non matched type 2 control patients are outlined in a previous study [20] (see table 1). However, as the non diabetic control group did not give informed consent we had very limited information regarding their clinical characteristics. When matched the average age of participants was 53 years (S.D. 15.6 yrs), 54 years (S.D. 20 yrs), and 54 years (S.D. 14.6 yrs) for the LADA, non-diabetic and type 2 diabetes controls respectively.

**Table 1:** Demographic characteristics

	LADA	Type 2 diabetes	Mean difference (95% CI)
Age (years)	49.6 ± 15.3	57.0 ± 14.8	7.4 (4.9-9.8)
BMI (kg/m^2^)	28.3 ± 5.7	31.3 ± 6.4	3.0 (1.9-4.0)

Patients with LADA (GAD antibody positive) had higher adiponectin levels than those with type 2 diabetes (GAD antibody negative) (Table 2). However, adiponectin levels for LADA patients did not differ from those found in non-diabetic controls.

**Table 2:** Adiponection in LADA Cases and the Controls

	LADA case (n = 64)	Type 2 control (n = 64)	Non-diabetic control (n = 64)	Mean difference LADA-Type 2 controls	Mean difference LADA-Nondiabetic controls
**Adiponectin** **(S.D)**	9.96 (6.1)	6.46 (5.0)	9.60 (6.3)	3.49 (95%CI: 1.7-5.3)	0.35 (95%CI: -1.8 to 2.5)
**Log transformed data**	0.919 (0.266)	0.717 (0.27)	0.892 (0.29)	0.20 (0.11-0.29) p = 0.0001	0.027 (-0.06 to 0.11) p = 0.54

**S.D-Standard Deviation**.

## Discussion

Patients with LADA have higher adiponectin levels than matched subjects with type 2 diabetes. This would suggest that abdominal obesity and visceral fat is less likely to be a risk factor or a causal factor for LADA. It is known that the BMI is lower among those with LADA compared with type 2 diabetes [20]. However, this study suggests that people with LADA are leaner for both abdominal/visceral fat as well as for subcutaneous/peripheral fat.

## Conclusions

This study confirms findings from other previous research [21] that the metabolic syndrome may not be a characteristic of LADA. It is possible that glycaemic control may be more of a risk factor for complications in LADA in the same way as classical Type 1 diabetes [15]. A prospective study with baseline adiponectin measurements is required within a sample of subjects with LADA to examine this further.

## List of abbreviations

LADA-latent autoimmune diabetes in adults, GADA-glutamic acid decarboxylase antibody, BMI-Body Mass Index.

## Competing interests

The authors have no conflicts of interest to disclose.

## Authors' contributions

All authors contributed to designing the research question and to writing the manuscript. SB, HD and RW collected the data, laboratory analysis was performed by SP and JL. Statistical analysis was performed by SB.
